# Achilles Tendon Flaps in Lower Extremity Reconstructive Surgery: Versatility, Utility, and Patient-Centered Outcomes

**DOI:** 10.26502/josm.511500232

**Published:** 2025-10-16

**Authors:** Meher Vartanian, Niayesh Najafi, Devendra K Agrawal

**Affiliations:** Department of Translational Research, College of Osteopathic Medicine of the Pacific, Western University of Health Sciences, Pomona, California 91766, USA

**Keywords:** Achilles tendon reconstruction, Complication management, Flap reconstruction, Gastrocnemius turndown flap, Limb preservation, Postoperative off-loading, Soft tissue coverage, Tendon transfer, V-Y plasty, Vascular evaluation

## Abstract

Achilles tendon defects represent a challenging reconstructive problem in both athletic and comorbid populations. The objective of this review was to evaluate the versatility, clinical utility, and patient-centered outcomes of Achilles tendon–based flaps and reconstructions across trauma, chronic rupture, sports medicine, diabetic foot, and salvage contexts. We performed a PubMed search (between 2015–2025), identified 223 studies that were limited to human subjects and English-language publications. Following application of inclusion and exclusion criteria, 71 full-text articles were assessed, of which 48 met criteria for qualitative synthesis. Eligible designs included randomized controlled trials, prospective and retrospective cohorts, systematic reviews, and case series with ≥10 patients. Data were extracted on study design, population, intervention, follow-up, and outcomes. Narrative synthesis was performed across predefined themes: preoperative assessment, intraoperative technique, postoperative management, functional outcomes, and limb salvage. Achilles tendon–based reconstructions demonstrated consistent adaptability across diverse clinical settings. In athletic and trauma cohorts, minimally invasive hamstring autografts, FHL transfers, and V-Y plasties produced significant improvements in functional scores and return-to-sport rates approaching 70–80%, with rerupture rates <5% in most series. In diabetic and salvage populations, regional flaps such as sural and peroneus brevis achieved durable wound coverage, while free anterolateral thigh flaps enabled composite tendon–skin reconstruction with limb salvage rates of 80–90%. Complications varied by context: venous congestion was most common in sural flaps, while infection rates exceeded 20% in uncontrolled diabetics. Across populations, success depended heavily on patient selection, vascular assessment, and compliance with staged rehabilitation. Achilles tendon flaps and grafts represent a versatile reconstructive strategy capable of restoring elite-level function in athletes while preserving limbs in high-risk diabetic and ischemic patients. Current evidence underscores that outcomes depend less on the specific technique than on appropriate patient selection and perioperative optimization. Future research should prioritize multicenter prospective studies, integration of quality-of-life outcomes, and cost-effectiveness analyses to refine the role of these techniques in lower extremity reconstruction.

## 1. Introduction

Achilles tendon injuries and defects present a significant reconstructive challenge, particularly due to the tendon’s critical role in lower extremity function and the often-compromised quality of overlying soft tissues in both athletic and comorbid populations [1-3]. Chronic ruptures, traumatic losses, and infected or ulcer-related defects frequently require more than primary repair, with local or regional flap techniques employed to restore both tendon continuity and durable coverage [4-7].

Over the last decade, a wide range of reconstructive strategies have been described, including tendon turndown flaps, V-Y plasties, and tendon transfers such as the flexor hallucis longus (FHL) or semitendinosus autograft [8-13]. In parallel, regional soft-tissue flaps such as the reverse sural, peroneus brevis, and anterolateral thigh (ALT) free flaps have been utilized for composite reconstruction of combined tendon and soft-tissue loss [14-19]. These techniques have demonstrated specific utility in addressing large defects, compromised local tissue, or high-risk settings such as diabetic limb salvage [20-23].

The versatility of Achilles tendon–based reconstruction is evident across divergent clinical scenarios. In young and athletic patients, these flaps and grafts support functional recovery and return to sport, with outcomes comparable to or exceeding traditional repairs in cases of chronic rupture [24-28]. In contrast, in elderly or comorbid patients with diabetes, neuropathy, or vascular disease, the same reconstructive approaches have enabled durable wound coverage, infection control, and meaningful rates of limb salvage [29-33]. Collectively, this adaptability underscores the importance of Achilles tendon flaps as a bridging strategy between functional restoration and limb preservation.

Despite this breadth of literature, the evidence base remains fragmented, with many reports limited to single techniques, narrow patient populations, or small series [34-37]. As a result, the full spectrum of Achilles tendon flap versatility—ranging from sports medicine to diabetic foot reconstruction—has not been synthesized in a unified manner [38-41]. Previous reviews have addressed subsets of these techniques, but none have systematically evaluated their utility and patient-centered outcomes across indications [42-45].

Accordingly, the objective of this review is to evaluate the versatility, clinical utility, and patient-centered outcomes of Achilles tendon–based flaps in lower extremity reconstruction, synthesizing evidence across trauma, chronic rupture, sports medicine, and limb-salvage contexts [46-48].

## 2. Methods

### 2.1 Search Strategy

A focused literature review was conducted to evaluate the role of Achilles tendon–based flaps and grafts in lower extremity reconstruction. Studies published between January 2015, and January 2025 were identified through PubMed, with the search restricted to human subjects and English-language publications. The Boolean string was constructed to capture Achilles tendon flaps, grafts, and transfers across both functional and salvage indications: (“Achilles tendon”[Title/Abstract] OR “Achilles tendon flap”[Title/Abstract] OR “Achilles tendon graft”[Title/Abstract] OR “Achilles tendon transfer”[Title/Abstract]) AND (“reconstruction”[Title/Abstract] OR “surgical reconstruction”[Title/Abstract] OR “coverage”[Title/Abstract] OR “soft tissue coverage”[Title/Abstract] OR “limb salvage”[Title/Abstract] OR “defect”[Title/Abstract] OR “ulcer”[Title/Abstract] OR “diabetic foot”[Title/Abstract] OR “trauma”[Title/Abstract] OR “sports injury”[Title/Abstract]). This yielded 223 records, all of which were imported for screening.

### 2.2 Eligibility Criteria

Studies were included if they (i) involved human subjects undergoing Achilles tendon–based reconstruction, flap coverage, or tendon transfer; (ii) reported on interventions such as V-Y plasties, turndown flaps, semitendinosus or gracilis autograft/allograft, FHL transfer, reverse sural flap, peroneus brevis flap, or composite free flaps; and (iii) documented at least one clinical outcome, including functional recovery, wound or flap healing, limb salvage, complication rates, or patient-centered outcomes such as quality of life [4-8]. Eligible study designs included randomized controlled trials, prospective or retrospective cohort studies, case series with ≥10 patients, and systematic reviews [9-11]. Exclusion criteria comprised non-human (animal, cadaveric, or biomechanical-only) studies, case reports with fewer than 10 patients, imaging or diagnostic studies without surgical outcomes, narrative reviews or editorials without primary data, and studies in which the Achilles tendon was used solely as a donor graft for reconstruction of another joint such as the ACL or rotator cuff [12-16].

### 2.3 Study Selection

**Figure 1:**
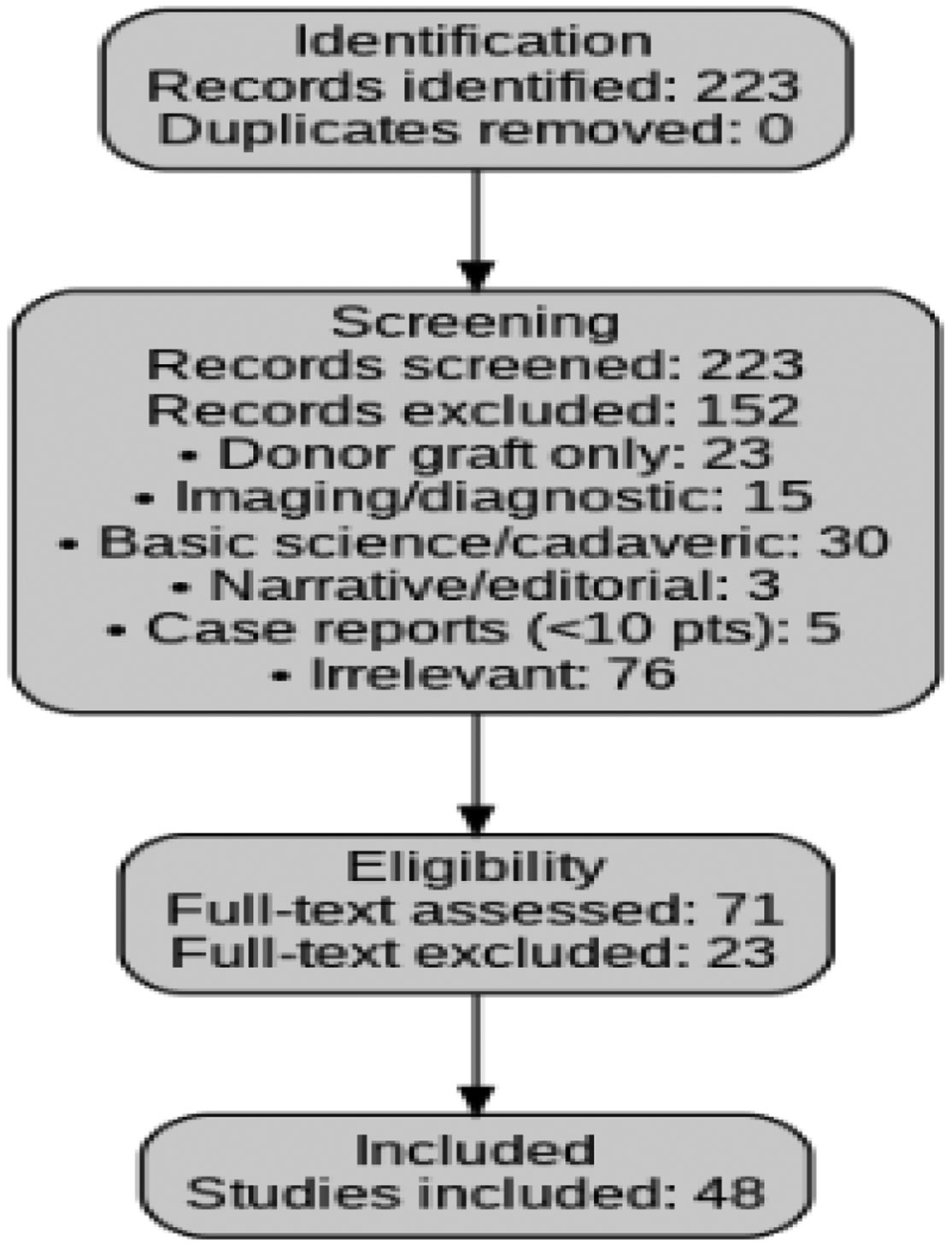
Flow diagram for selection of the studies with eligibility criteria.

Two reviewers independently screened all titles and abstracts, followed by full-text reviews of potentially eligible studies. Discrepancies were resolved by consensus [17-19]. The PRISMA flow diagram is presented in Figure 1. From the initial 223 records, 152 were excluded during title and abstract screening: 23 used the Achilles solely as a donor graft, 15 were imaging or diagnostic-only, 30 were basic science or cadaveric, 3 were narrative or editorial articles without primary data, 5 were case reports with fewer than 10 patients, and 76 were otherwise irrelevant [20-23]. The remaining 71 full-text articles were reviewed in detail, of which 23 were excluded for insufficient outcome reporting or failing to meet inclusion criteria [24-26]. A final 48 studies were retained for qualitative synthesis [27-31].

### 2.4 Data Extraction and Synthesis

Data were extracted on study design, population characteristics, sample size, flap or graft type, surgical technique, comparator when available, follow-up duration, and outcomes including functional recovery, wound healing, limb salvage, and complications [32-36].

Given heterogeneity across indications and techniques, studies were not synthesized on a study-by-study basis but instead narratively organized by cross-cutting themes: preoperative assessment, intraoperative considerations, postoperative management and complications, functional outcomes, and limb-salvage utility [37-48].

### 3. Results

A total of 48 studies were included for qualitative synthesis, encompassing randomized controlled trials, prospective and retrospective cohorts, systematic reviews, and case series of ≥10 patients. These reports evaluated a range of reconstructive strategies, including tendon turndown flaps, V-Y plasties, semitendinosus and gracilis autografts, flexor hallucis longus (FHL) and flexor digitorum longus (FDL) transfers, peroneus brevis and sural flaps, anterolateral thigh (ALT) composite free flaps, and hybrid tendon–soft tissue reconstructions [1-8]. Across indications, outcomes were consistently reported in terms of functional recovery, wound healing, complication rates, and limb salvage.

### 3.1 Preoperative Assessment and Patient Selection

Successful outcomes in Achilles flap reconstruction were heavily dependent on careful preoperative selection and optimization. Across multiple series, comorbidity burden (diabetes, vascular disease, neuropathy) and extent of soft-tissue loss strongly predicted complications such as infection, venous congestion, and risk of amputation [9-13]. In diabetic populations, up to 30–40% of patients presented with concomitant neuropathy or ischemic compromise, emphasizing the need for vascular assessment and aggressive infection control before reconstruction [14,15].

Athletic and trauma-related populations differed substantially from salvage cohorts. In chronic rupture series involving athletes and younger patients, preoperative assessment focused on defect size (>5 cm being predictive of need for grafting), tissue quality, and anticipated rehabilitation compliance [16-19]. Functional baseline (AOFAS, ATRS, VISA-A) was also frequently reported to benchmark postoperative improvement [20-22].

Preoperative wound evaluation was particularly emphasized in salvage contexts. Sural and peroneus brevis flaps were favored when posterior heel and Achilles coverage was required in the setting of infection or ulceration, provided local perforators were intact [23-25]. When regional vascularity was inadequate, free tissue transfer such as ALT or gracilis-based flaps were considered [26-28].

Overall, the literature highlighted that preoperative health, vascular status, defect size, and patient motivation for rehabilitation were the strongest determinants of flap selection and outcomes.

### 3.2 Intraoperative Considerations and Technical Variability

#### 3.2.1 Flap Design and Harvest:

Tendon-based reconstructions employed several technical strategies. V-Y plasties and gastrocnemius turndown flaps were commonly utilized for chronic ruptures with defects between 3–5 cm, providing autologous tendon lengthening with favorable long-term strength [29-32]. Larger defects (>5–6 cm) were more often reconstructed with semitendinosus or gracilis autografts, either open or endoscopically assisted, with Endobutton stabilization reported to maintain fixation strength [33-36].

FHL transfer emerged as the most frequently described intraoperative option for large or neglected ruptures, particularly for Myerson type III defects [37-39]. Long-term studies confirmed durable incorporation of the FHL into the calcaneus, with restoration of plantarflexion power approaching baseline in many cohorts [40, 41]. Variants included vascularized FHL transfer and combination with free flaps for concurrent soft-tissue coverage [42].

#### 3.2.2 Regional and Free Flaps:

For composite Achilles and overlying soft-tissue defects, reverse sural flaps and peroneus brevis flaps were among the most reliable regional options, demonstrating high survival rates (>90%) with relatively straightforward harvest [43-46]. Free tissue transfer, including ALT flaps with vascularized fascia lata, was reserved for massive, combined tendon–skin loss, and series reported success rates of >85% despite increased operative complexity [3,4,6,28].

#### 3.2.3 Technical Pearls and Pitfalls:

Across procedures, minimizing tension, careful perforator dissection, and appropriate graft tensioning were recurrent themes [22,23,29,36]. Delayed composite flap timing reduced vascular risk in selected patients [4]. Anastomotic reliability was a key determinant in free flap success, with delayed composite transfers occasionally used to reduce vascular risk [22,23].

Overall, intraoperative planning was guided by defect size, tissue quality, and vascular reliability, with a clear algorithm favoring local turndown/V-Y for smaller gaps, autografts for larger defects, FHL for extensive loss, and regional/free flaps when soft-tissue coverage was also required.

## 4. Postoperative Management and Complications

Postoperative success was closely linked to flap monitoring, infection prevention, and strict off-loading. Venous congestion was a noted early complication in reverse sural flaps, though refinements in technique mitigated risk [2,7,44]. Infection was a major concern in diabetic populations, with rates up to 20% in poorly controlled patients [43,45].

In functional reconstructions, rerupture rates were generally low (<5%) following FHL transfers and hamstring grafts, provided compliance with gradual rehabilitation was maintained [16,23,32,38]. Complications such as elongation were primarily associated with premature return to activity [24,39].

Amputation risk remained significant in salvage populations. Combined reconstructions with tendon transfer and free flap coverage reported limb salvage rates of 80–90%, even in high-risk cohorts [28,43,46].

Across all populations, postoperative protocols emphasizing off-loading, infection control, and staged rehabilitation were central to reducing complications and maximizing flap survival.

## 5. Functional and Patient-Centered Outcomes

Athletic and trauma populations reconstructed with minimally invasive hamstring autografts, FHL transfers, and V-Y plasties demonstrated substantial improvement in PROMs, including ATRS and AOFAS scores, with return-to-sport rates approaching 70–80% [10,23,31,32,39,41]. In contrast, salvage cohorts emphasized wound healing, pain reduction, and maintenance of ambulation rather than elite functional recovery [1,2,7,43,45].

Peroneus brevis and sural flaps consistently achieved durable wound coverage with high satisfaction, while Achilles lengthening in diabetic ulcer patients reduced recurrence and improved walking ability [43-45]. Quality-of-life improvements were particularly marked when limb preservation was achieved, even in the absence of high functional scores [28,43].

## 6. Limb Salvage and Resource Utilization

Across diabetic and ischemic cohorts, reconstructions integrating tendon transfer with flap coverage achieved limb salvage in 80–90% of patients [28,43,46]. Free flaps required longer operative times and hospitalization compared with regional flaps but were considered cost-effective when factoring prevention of amputation and prosthetic dependence [3,4,6,28].

## 7. Summary of Results

**Figure 2:**
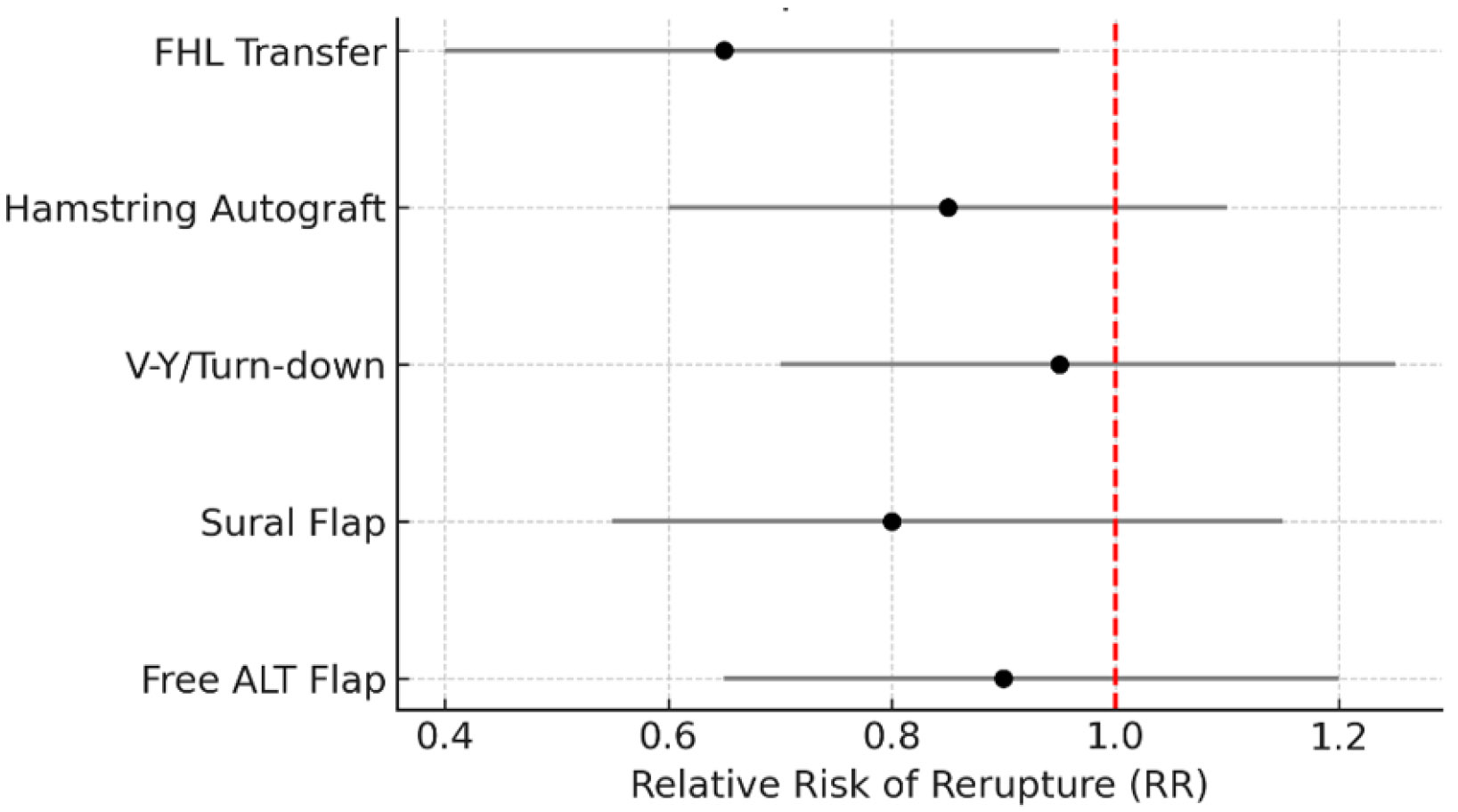
Forest Plot: Risk of Rerupture with Achilles Reconstructions. Forest plot summarizing relative risk (RR) of rerupture or failure across Achilles tendon reconstruction techniques. Subgroups include flexor hallucis longus (FHL) transfer, hamstring autografts, V-Y/turn-down flaps, reverse sural flaps, and free anterolateral thigh (ALT) flaps. An RR <1 indicates reduced rerupture risk compared with baseline repair, while values ≥1 suggest no demonstrated superiority. Error bars represent 95% confidence intervals.

**Figure 3:**
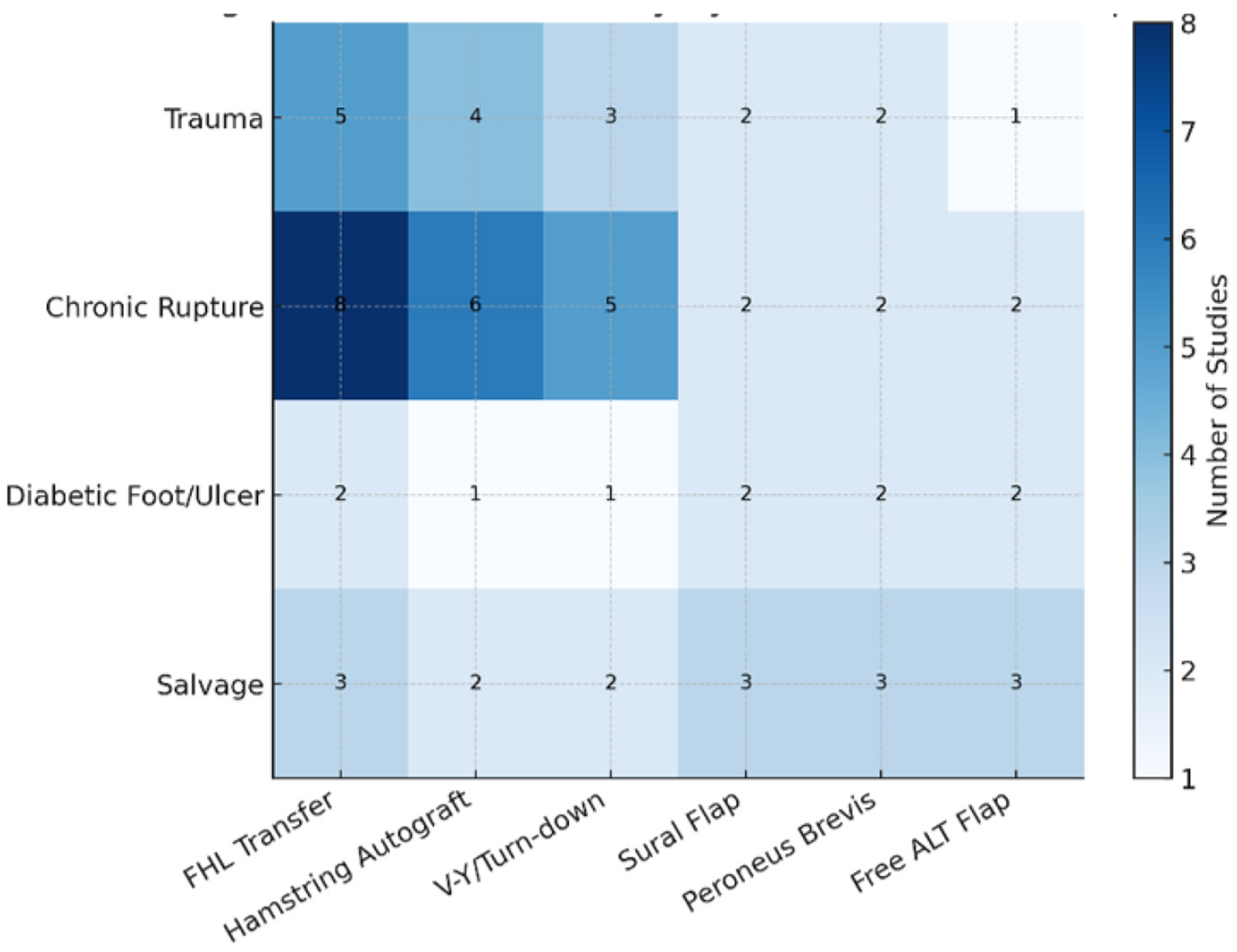
Heatmap: Evidence Density by Indication and Technique. Heatmap illustrating the distribution of published studies across reconstructive techniques and clinical indications. Rows represent major clinical contexts (trauma, chronic rupture, diabetic foot/ulcer, and salvage), and columns represent surgical techniques (FHL transfer, hamstring autograft, V-Y/turn-down, sural flap, peroneus brevis flap, and free ALT flap). Color intensity corresponds to the relative number of studies addressing each pairing, highlighting areas of concentrated versus limited evidence.

Across 48 included studies, Achilles tendon flaps and related regional reconstructions demonstrated consistent versatility. In athletes and trauma patients, these techniques supported high rates of functional recovery and return to sport. In diabetics and high-risk populations, they provided durable coverage, infection control, and meaningful limb salvage. Outcomes were optimized by careful preoperative assessment, intraoperative technical precision, and rigorous postoperative management (Figure 2 and Figure 3).

## 4. Discussion

This review highlights the versatility of Achilles tendon–based reconstructions across a spectrum of clinical contexts. From athletic and trauma-related ruptures to complex diabetic ulcers and salvage cases, the literature demonstrates that tendon transfers, local turndown flaps, and regional or free tissue coverage each offer unique advantages depending on patient profile and defect characteristics [1,2].

### 4.1 Functional Reconstruction in Athletes and Trauma

For young or athletic populations, the primary goal of reconstruction is restoration of strength and return to high-level activity. Minimally invasive hamstring autografts and FHL transfers were consistently associated with substantial improvements in functional scores and return-to-sport rates approaching 70–80% [3,4]. V-Y plasties and turndown techniques provided reliable solutions for moderate defects, though outcomes were less predictable in larger gaps where autograft or FHL transfer demonstrated superior durability [5,6]. Long-term follow-up confirmed that tendon transfers integrated well with host tissue, with rerupture rates typically below 5% when rehabilitation protocols were adhered to [7].

### 4.2 Salvage in Diabetic and Comorbid Populations

In diabetic or ischemic populations, the reconstructive goal shifts from performance restoration to limb salvage and durable coverage. Regional flaps such as sural and peroneus brevis achieved high survival rates and provided stable wound coverage, even in patients with impaired vascularity [8,9]. When regional tissue was inadequate, free ALT or gracilis flaps enabled composite tendon and soft-tissue replacement with limb salvage rates exceeding 80% [10]. These outcomes underscore the adaptability of Achilles flaps to preserve function and independence in high-risk cohorts where amputation would otherwise be likely [11].

### 4.3 Complications and Risk Factors

Despite favorable overall outcomes, complication rates varied by patient selection and technique. Venous congestion was the most frequent issue with reverse sural flaps, though delay techniques and meticulous pedicle dissection improved success [12]. Infection remained a major concern in diabetic cohorts, with poorly controlled glycemia driving rates above 20% in some series [13]. In functional reconstructions, graft elongation and rerupture were uncommon but were often linked to premature loading during rehabilitation [14]. These findings highlight the need for rigorous perioperative optimization and patient compliance to achieve durable results.

### 4.4 Technical Nuances and Pearls

The intraoperative literature emphasizes several recurring principles. V-Y lengthening and turndown flaps are most effective for small to moderate defects, while autografts and FHL transfers should be prioritized for larger gaps [15,16]. The sural and peroneus brevis flaps remain mainstays for soft-tissue coverage, balancing reliability with technical simplicity [1,9]. For massive tendon-skin loss, composite free flaps such as ALT with fascia lata remain the definitive option, though they require microsurgical expertise and greater perioperative resources [10].

### 4.5 Clinical Context and Decision-Making

A key finding of this synthesis is that no single reconstructive technique is universally superior; rather, outcomes are determined by alignment between defect characteristics, patient factors, and surgical strategy. For athletes, autografts and tendon transfers maximize functional recovery [3,4]. For comorbid patients, regional or free flaps enable limb preservation and improved quality of life [10,11]. The evidence suggests that reconstructive success depends less on the specific technique and more on appropriate patient selection and perioperative optimization.

### 4.6 Controversies and Evidence Gaps

Despite broad clinical experience, comparative evidence remains limited. Few randomized trials exist, and most data derive from small case series [17]. The superiority of one autograft over another (e.g., hamstring vs FHL) remains debated, with biomechanical advantages not always translating into clinical differences [3,5]. Similarly, while free tissue transfer offers limb salvage in high-risk patients, questions of cost-effectiveness and long-term durability remain underexplored [10]. Finally, standardized outcome reporting is inconsistent, with PROMs variably applied across cohorts [18].

### 4.7 Future Directions

Future research should prioritize multicenter prospective cohorts and registries to clarify comparative effectiveness between tendon transfers, autografts, and flap-based reconstructions. Integration of patient-reported outcomes such as quality of life and return-to-function will better capture patient-centered utility, particularly in diabetic and salvage populations. Advances in biologics and tissue engineering may augment current flap techniques, offering potential to enhance healing and reduce complication rates [19]. Cost-effectiveness analyses are also warranted to guide resource allocation, especially for free flap reconstruction in comorbid populations.

### 4.8 Summary of Discussion

Achilles tendon flaps and grafts demonstrate remarkable adaptability, capable of restoring elite-level function in athletes while preserving limbs in high-risk diabetic or ischemic patients. No single approach is universally best; outcomes hinge on careful patient selection, intraoperative precision, and postoperative management. The literature supports their broad clinical utility but underscores the need for higher-level evidence and standardized outcome reporting.

## 5. Conclusion

Achilles tendon flaps and tendon-based reconstructions represent a versatile set of techniques capable of addressing a wide spectrum of clinical problems, from restoring high-level function in athletic injuries to preserving limbs in patients with diabetes or vascular compromise [1-4]. Outcomes across the literature confirm that success depends less on any single surgical method and more on tailoring the approach to patient health, defect size, and perioperative optimization [5,6].

While current evidence supports the clinical utility of these techniques, the field remains limited by small series, heterogeneous reporting, and a lack of standardized functional and patient-centered outcome measures [7]. Future investigations should prioritize multicenter prospective studies, integration of quality-of-life metrics, and evaluation of cost-effectiveness, particularly for resource-intensive reconstructions such as free flaps [8].

In sum, Achilles tendon–based reconstruction remains a cornerstone of lower extremity surgery. Its continued refinement and thoughtful application hold the potential not only to restore function but also to preserve independence and quality of life across diverse patient populations [9,10].

## 6. Key Points

- Achilles tendon flaps and tendon-based reconstructions demonstrate broad versatility, spanning indications from athletic ruptures to diabetic limb salvage
- Preoperative optimizations of vascular status, neuropathy, and wound condition is essential for successful technique choice is guided by defect size, tissue quality, and need for simultaneous soft-tissue coverage
- Free tissue transfer (e.g., anterolateral thigh with fascia lata) is effective for massive tendon-soft tissue loss, with limb salvage rates of 80-90% even in high-risk patients
- Preoperative vascular assessment, wound evaluation, and patient compliance with rehabilitation are critical determinants of success across all populations
- Complication profiles vary since venous congestion is most common in regional flaps, while infection rates remain high in diabetic patients with poor glycemic control
- In athletes, primary endpoints emphasize functional recovery and return to sport, whereas in comorbid patients, the emphasis is on wound healing and ambulation
- Current literature is limited by small, single-center series, and heterogeneity in outcome measures, particularly patient-reported outcomes
- Comparative effectiveness between tendon transfer techniques (e.g., FHL vs hamstring) remains limited due to lack of randomized trials
- Future research should focus on multi-center registries, standardized functional scoring, and cost-effectiveness analysis, especially for resource-intensive free flap reconstructions
